# Outcome of cerebral thrombectomy cannot be predicted by systolic function as long as not all influencing factors are analyzed

**DOI:** 10.1590/1806-9282.20260414

**Published:** 2026-08-07

**Authors:** Carla Alexandre Scorza, Fulvio Scorza, Josef Finsterer

**Affiliations:** 1 Universidade Federal de São Paulo, Escola Paulista de Medicina – São Paulo (SP), Brazil.; 2 Neurology & Neurophysiology Censer – Vienna, Austria.

Dear Editor,

We read with interest the article by Ertuğrul et al. on a retrospective study investigating the relationship between systolic function (assessed by transthoracic echocardiography [TTE]) and treatment outcome in patients with ischemic stroke following mechanical thrombectomy. The study aimed to identify predictors of poor prognosis^1^. The 144 patients with unsuccessful recanalization were older, had a higher NIHSS (National Institutes of Health Stroke Scale) score, a longer time from puncture to recanalization, a lower ASPECT (Alberta Stroke Program Early Computed Tomography) score, a lower ejection fraction, and a higher left ventricular end-diastolic diameter than the 176 patients with successful recanalization^1^. The authors concluded that an unfavorable outcome after mechanical thrombectomy is associated with a low ASPECT score, a high NIHSS score, and reduced systolic function^1^. The study is interesting, but some points should be discussed.

The first point concerns the retrospective study design^1^. Retrospective studies have several disadvantages, such as low data quality, missing data, the inability to demonstrate causal relationships, susceptibility to memory and selection biases, difficulties in controlling for confounding factors, and generally lower levels of evidence compared to prospective studies^2^. Retrospective studies cannot demonstrate temporal relationships as clearly as prospective studies, which further complicates the demonstration of causal relationships^2^.

The second point is that no echocardiography was performed before the procedure^1^. Therefore, a comparison of echocardiographic parameters before and after the procedure cannot be made. Since it cannot be ruled out that the echocardiographic findings were already present before the stroke and may even have contributed to its development, it cannot be excluded that the reported results are not related to the outcome of the thrombectomy.

The third point is that the cardiac response to thrombectomy can vary significantly depending on the type of cerebral artery treated^3^. The cardiac response may differ depending on whether the thrombus was removed from the middle cerebral artery, the basal artery, the posterior cerebral artery, or the internal carotid artery. Therefore, it is recommended to review the echocardiographic findings to determine if there were any differences between the vascular territories.

The fourth point is that the echocardiographic results can also depend on the latency period of the TTE after the procedure. Since TTEs could be performed up to 3 days post-procedure, according to the methods section, there is considerable heterogeneity in latency periods between patients. Because TTE results can be highly dependent on latency, it is conceivable that the TTE was influenced by numerous other factors that arose only after the procedure. These include infections, comorbidities, and nutritional and hydration status.

The fifth point concerns the medications the patient received after thrombectomy. These were not included in the analysis. Since various medications and comorbidities can impair systolic function^4^, it would have been crucial to include any concomitant medications and comorbidities that arose after the thrombectomy in the analysis.

Finally, it should be clarified whether the echocardiography was performed by the same examiner in all examinations. If different examiners performed the TTE, the interobserver variability must be reported.

Unless all factors influencing systolic function are included in the analysis, reduced systolic function cannot be recommended as a predictor of the success of cerebral thrombectomy.

## Data Availability

The datasets generated and/or analyzed during the current study are available from the corresponding author upon reasonable request.
